# Horseradish Peroxidase-Encapsulated Fluorescent Bio-Nanoparticle for Ultra-Sensitive and Easy Detection of Hydrogen Peroxide

**DOI:** 10.3390/bios13020289

**Published:** 2023-02-17

**Authors:** Myeong-Jun Lee, Ji-Ae Song, Jin-Ha Choi, Jeong-Hyeop Shin, Ji-Woon Myeong, Ki-Ppeum Lee, Taehwan Kim, Ki-Eob Park, Byung-Keun Oh

**Affiliations:** 1 Department of Chemical and Biomolecular Engineering, Sogang University, Seoul 04107, Republic of Korea; 2 School of Chemical Engineering, Jeonbuk National University, Jeonju-si 54896, Republic of Korea; 3 UNIANCE Inc., Seongnam-si 13403, Republic of Korea

**Keywords:** gold nanocluster, hydrogen peroxide, fluorescence sensor, point-of-care test, bio-nanoparticles

## Abstract

Hydrogen peroxide (H_2_O_2_) has been a fascinating target in various chemical, biological, clinical, and industrial fields. Several types of fluorescent protein-stabilized gold nanoclusters (protein-AuNCs) have been developed for sensitive and easy detection of H_2_O_2_. However, its low sensitivity makes is difficult to measure negligible concentrations of H_2_O_2_. Therefore, to overcome this limitation, we developed a horseradish peroxidase-encapsulated fluorescent bio-nanoparticle (HEFBNP), comprising bovine serum albumin-stabilized gold nanoclusters (BSA-AuNCs) and horseradish peroxidase-stabilized gold nanoclusters (HRP-AuNCs). The fabricated HEFBNP can sensitively detect H_2_O_2_ owing to its two properties. The first is that HEFBNPs have a continuous two-step fluorescence quenching mechanism, which comes from the heterogenous fluorescence quenching mechanism of HRP-AuNCs and BSA-AuNCs. Second, the proximity of two protein-AuNCs in a single HEFBNP allows a reaction intermediate (•OH) to rapidly reach the adjacent protein-AuNCs. As a result, HEFBNP can improve the overall reaction event and decrease the loss of intermediate in the solution. Due to the continuous quenching mechanism and effective reaction event, a HEFBNP-based sensing system can measure very low concentrations of H_2_O_2_ up to 0.5 nM and show good selectivity. Furthermore, we design a glass-based microfluidic device to make it easier use HEFBNP, which allowed us to detect H_2_O_2_ with the naked eye. Overall, the proposed H_2_O_2_ sensing system is expected to be an easy and highly sensitive on-site detection tool in chemistry, biology, clinics, and industry fields.

## 1. Introduction

Hydrogen peroxide (H_2_O_2_) is a relatively stable reactive oxygen species (ROS), playing an important role in oxidative damage, redox signaling, and maintaining the physiological balance of organisms in various fields, such as chemistry, biology, clinics, and industry [1,2]. In addition, many studies have attempted to precisely detect H_2_O_2_ to track metabolic reactions because H_2_O_2_ is a byproduct of enzymatic reactions [3,4]. For many reasons, precise detection of H_2_O_2_ is of great importance; however, H_2_O_2_ could exist in negligible concentrations when analyzed. For example, H_2_O_2_ was present at a negligible concentration of 20~800 nM on the ocean surface [5]. So, various fields have focused on a highly sensitive sensing technology in order for the precise detection of H_2_O_2_. 

For precise and sensitive detection of H_2_O_2_, many technologies have been used as sensing methods, such as fluorescence, electrochemistry, chemiluminescence, spectrophotometry, and colorimetry [6]. Among various technologies, fluorescence-based H_2_O_2_ sensors have been used because of their simplicity, high sensitivity, and fast detection [7]. In recent years, gold nanoclusters (AuNCs) have gained significant attention in H_2_O_2_ fluorescence sensing owing to their advantages such as a significant Stokes shift, considerable photostability, long fluorescence lifetime, and high emission rate [8]. AuNCs are ultra-small particles composed of hundreds of gold atoms that lead to molecule-like properties, such as catalytic activity and strong luminescence [9]. As AuNCs are unstable in solution, it was necessary to stabilize them using ligands such as dendrimers, peptides, DNA, and proteins [10,11]. Among the many types of AuNCs, protein-stabilized AuNCs (protein-AuNCs) have attractive advantages such as good biocompatibility, bioactivity, eco-friendly production processes, and stability over a wide pH range because they can integrate the properties of proteins and AuNCs into a single entity [12]. In particular, protein-AuNCs have been used to measure H_2_O_2_ because of their distinct optical properties, in that the protein-AuNCs’ fluorescence was quenched by interaction with H_2_O_2_ [13]. Owing to these unique properties, AuNCs stabilized by several types of proteins have been used for fluorescence detection of H_2_O_2_. For example, Jain et al. reported that bovine serum albumin-stabilized gold nanoclusters (BSA-AuNCs) can detect H_2_O_2_ within a linear range from 1 μM to 50 mM with a limit of detection (LOD) of 700 nM [14]. Wen et al. developed horseradish peroxidase-stabilized gold nanoclusters (HRP-AuNCs) for the sensitive detection of H_2_O_2_ and exhibited a linear response in the range of 100 nM to 100 μM with an LOD of 30 nM [15]. However, these values are difficult to measure at negligible concentrations of H_2_O_2_. Therefore, it is necessary to develop a technology for the highly sensitive detection of H_2_O_2_.

To overcome sensitivity limitations, we newly designed a horseradish peroxidase-encapsulated fluorescent bio-nanoparticle (HEFBNP), comprising BSA-AuNCs and HRP-AuNCs. The HEFBNPs could sensitively detect H_2_O_2_ because of their two distinctive properties, arising from heterogeneous mixing proteins in one particle. The first property is their continuous two-step fluorescence quenching effect that contributes to the high fluorescence quenching effect of HEFBNP. The interaction of H_2_O_2_ and HRP-AuNCs produced •OH. With this reaction, the fluorescence of HRP-AuNCs was quenched by the deformation of AuNCs [16,17]. Subsequently, •OH, which has strong oxidative power, catalyzed the fluorescence quenching of AuNCs in HRP and BSA [18,19]. Therefore, one H_2_O_2_ molecule could quench the fluorescence of two or more AuNCs, resulting in HEFBNPs effectively detecting H_2_O_2_ compared to single protein-AuNCs. The second property is the close distance of HRP-AuNCs, which by crosslinking improved the overall reaction event for the sensitive detection of H_2_O_2_. In the continuous reaction, the overall reaction event was determined by how fast •OH, which is an intermediate, reached the adjacent protein-AuNCs. In HEFBNPs, owing to the proximity of the two protein-AuNCs in single HEFBNPs, •OH was rapidly delivered to the adjacent protein-AuNCs, thus enhancing the overall reaction event, with •OH loss decreased in the solution [20]. In addition, HEFBNPs encapsulated several enzymes and AuNCs in a single nanoparticle that enhanced the catalytic reaction. These unique properties of HEFBNPs enabled the detection of H_2_O_2_ with an improved fluorescence quenching effect.

In this study, we demonstrate the synthesis method and characteristics of HEFBNPs. In addition, we demonstrate a highly sensitive fluorescence biosensor for detecting H_2_O_2_ using the excellent quenching properties of the HEFBNPs in the presence of H_2_O_2_. Moreover, for the convenient detection of H_2_O_2_ using HEFBNPs, we fabricated a glass-based microfluidic device to visually detect the concentration of H_2_O_2_. Owing to the lightweight simple detection method and short response time of the device, our sensing platform was very suitable for application in a point-of-care test (POCT) for detecting H_2_O_2_.

## 2. Materials and Methods

### 2.1. Materials

Bovine serum albumin (BSA), glutaraldehyde, gold (III) chloride trihydrate (HAuCl_4_. 3H_2_O), potassium chloride (KCl), potassium bromide (KBr), sodium hydroxide (NaOH), magnesium chloride (MgCl_2_), sodium bicarbonate (NaHCO_3_), boric acid (H_3_BO_3_), Zinc chloride (ZnCl_2_), iron (II) chloride (FeCl_2_), iron (III) chloride (FeCl_3_), sodium hypochlorite solution (NaClO), and 3,3′,5,5′-Tetramethylbenzidine (TMB) were purchased from Sigma Aldrich (St. Louis, MO, USA). Acetonitrile (C_2_H_3_N), 30% hydrogen peroxide (H_2_O_2_), and pure ethanol (C_2_H_5_OH) were purchased from Daejung Chemicals (Siheung, Republic of Korea). Sodium chloride (NaCl), calcium chloride dihydrate (CaCl_2_·2H_2_O), and sodium sulfate (Na_2_SO_4_) were purchased from Junsei (Tokyo, Japan). Horseradish peroxidase (HRP) was purchased from Thermo Fisher Scientific (Waltham, MA, USA). Distilled water (DW) was purified using a Milli-Q system (Darmstadt, Germany).

### 2.2. Apparatus

Transmission electron microscopy (TEM) images were obtained using a JEOL1010 microscope (Tokyo, Japan). For the TEM sample preparation, the synthesized gold nanoclusters were dried on a carbon-coated copper grid at 24 °C. Dynamic light scattering (DLS) analysis was performed using a zeta sizer (USA). The excitation and emission wavelengths of the BSA-AuNCs, HRP-AuNCs, and HEFBNP were recorded by 3D scanning using an F-7000 fluorescence spectrophotometer (HITACHI, Tokyo, Japan) to investigate the optical properties. The emission wavelength was scanned from 500 to 800 nm as the excitation wavelength was increased stepwise from 200 to 500 nm. Absorption spectra were obtained using a NanoDrop spectrophotometer (Thermo Scientific, Waltham, MA, USA). All the fluorescence spectra were acquired using an F-7000 fluorescence spectrophotometer. The excitation and emission slit widths were maintained at 10 nm/s, and the response time was 2400 nm/s, respectively. The physical fluorescence under the UV light was observed using a UV illuminator (MDM, Seoul, Korea).

### 2.3. Synthesis of BSA-AuNCs and HRP-AuNCs

Protein-stabilized gold nanoclusters (BSA-AuNCs and HRP-AuNCs) were synthesized using a previously reported method [21,22]. Briefly, 500 μL of protein (BSA or HRP) solution (100 mg/mL) was mixed with an equal volume of HAuCl4 solution (10 mM) and incubated at 37 °C for 5 min with vigorous stirring (800 rpm). Next, 50 μL of NaOH solution (1 M) was added to the aqueous solution and incubated at 37 °C with stirring for 24 h. The synthesized BSA-AuNCs and HRP-AuNCs were stored in the dark at 4 °C until further use.

### 2.4. Synthesis of HEFBNPs

HEFBNPs were synthesized by the desolvation method and crosslinking of the BSA-AuNC and HRP-AuNC mixture [23]. First, 100 μL BSA-AuNCs (50 mg/mL) was mixed with 10 μL HRP-AuNCs (50 mg/mL). Next, a dissolving agent composed of acetonitrile and 100% ethanol in a ratio of 4:1 was added dropwise to the mixture under vigorous stirring at 12 °C. During the dissolution process, the solubility of BSA-AuNCs and HRP-AuNCs decreased, and the mixture turned opaque [24]. For enabling crosslinking between BSA-AuNCs and HRP-AuNCs, 40 μL of 1% glutaraldehyde was added to the solution and incubated at 4 °C for 2 h. During the crosslinking reaction, new C=N bonds were formed between the amino groups of the protein to maintain a constant shape [25,26,27,28]. After the reaction was complete, the excess chemicals were removed by centrifugation at 9500 rpm at 12 °C for 15 min. The supernatant was discarded, and the pellet was dispersed in 200 μL of DW. The washing step was repeated more than three times. At the final washing step, the supernatant was removed, and 100 μL of DW was added to the pellet. The synthesized HEFBNPs were stored in the dark at 4 °C (Figure S1, in the Supplementary Materials). The non-fluorescence horseradish peroxidase-encapsulated bio-nanoparticles (HEBNPs), which were composed of BSA and HRP, were synthesized by same procedure.

### 2.5. Experiments of H_2_O_2_ Sensing Using HEFBNPs

**Scheme 1:**
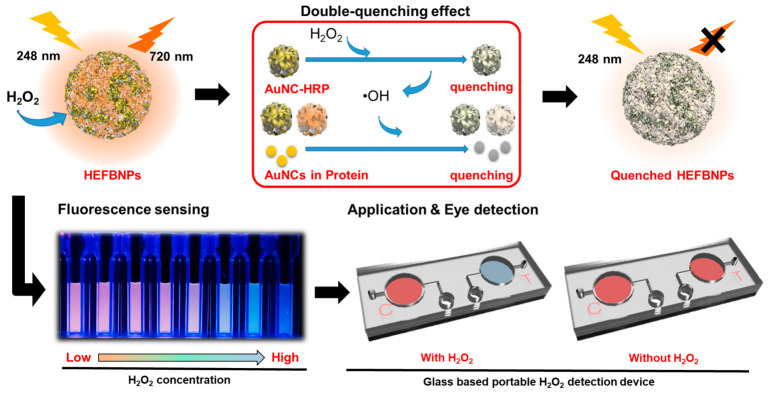
Schematic illustration of the fluorescence quenching mechanism of HEFBNPs by addition of H_2_O_2_ and its applications. (•OH = hydroxyl radical).

H_2_O_2_ detection using HEFBNPs is illustrated in Scheme 1. We used three experimental methods to observe the variation in fluorescence according to the concentration of H_2_O_2_. First, a photograph of fluorescence under UV light was obtained using a UV illuminator for visual detection. Second, fluorescence spectra were recorded using an F-7000 fluorescence spectrophotometer to obtain more detailed and quantitative concentrations of H_2_O_2_. In addition, the fluorescence quenching effect of HEFBNPs was compared to that of BSA-AuNCs and HRP-AuNCs by recording fluorescence spectra under identical conditions. The selectivity toward H_2_O_2_ was evaluated by investigating the response of HEFBNPs to other components. Third, using the fabricated glass-based microfluidic device, the fluorescence change of the HEFBNPs was investigated with the naked eye. 

## 3. Results and Discussion

### 3.1. Size and Morphology of HEFBNPs

**Figure 1:**
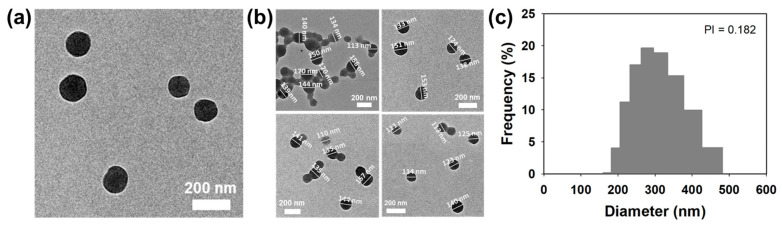
(**a**) Morphology of HEFBNPs: TEM image, (**b**) particle size distribution according to various HEFBNPs in TEM image (average = 134 ± 15 nm), and (**c**) particle size distribution analysis using DLS analysis.

The size and morphology of the synthesized HEFBNPs were investigated by TEM and DLS. TEM images showed that the synthesized HEFBNPs had a spherical structure (Figure 1a). To measure the statistic diameter of HEFBNPs, TEM images of HEFBNPs were analyzed by ‘Image J’. From those results, we could figure out that the average diameter of HEFBNPs was 134 ± 15 nm (Figure 1b). DLS analysis was also conducted to investigate the particle size of HEFBNPs, with the results shown in Figure 1c. The size of the HEFBNPs was distributed in the range of 200~400 nm, with an average diameter of 300 nm (PI = 0.182). A comparison of the results from TEM and DLS data showed that the sizes of the HEFBNPs were negligibly different. The differences in the sizes of HEFBNPs measured by the TEM and DLS results are explained by the principle of analysis techniques. DLS was used to measure the hydrodynamic radius of the dispersed particles associated with the ionic and solvent layers. However, the TEM images show that the particles shrunk during the drying process [29]. The presence or absence of HRP-AuNCs within the HEFBNP was examined as a TMB color change by enzyme reaction (Figure S2). From these results, we could claim that HEFBNPs were well synthesized by desolvation and crosslinking processes with the HRP-AuNC and BSA-AuNC mixture.

### 3.2. Fluorescence Properties of HEFBNPs

**Figure 2:**
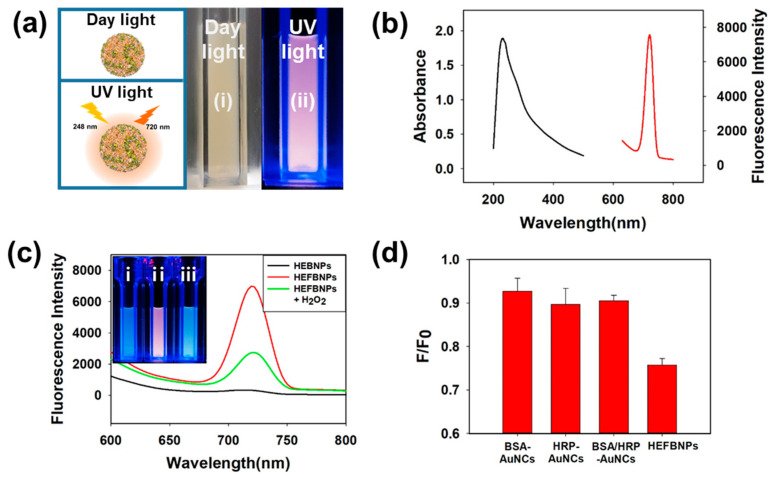
(**a**) Image of HEFBNPs (**i**) color under daylight and (**ii**) fluorescence under UV light. (**b**) Fluorescence absorbance and emission spectrum. (**c**) Fluorescence spectrum of (**i**) HEBNPs and HEFBNPs (**ii**) in the presence and (**iii**) absence of H_2_O_2_. (**d**) Comparison of fluorescence intensity of HEFBNPs, BSA-AuNCs, HRP-AuNCs, and mixture of BSA-AuNCs and HRP-AuNCs in the presence of H_2_O_2_.

When the protein-AuNCs absorbed UV light, they showed their own fluorescence depending on the type of protein or metal nanocluster. Photographs of the HEFBNP solution under visible light and UV light are shown in Figure 2a. The HEFBNPs showed an almond color under daylight (Figure 2a(i)). However, HEFBNPs showed a strong reddish-orange fluorescence under UV light (Figure 2a(ii)). HEFBNPs’ excitation and emission wavelengths were studied by 3D scanning (data were not shown) to further investigate their fluorescence properties, such as absorbance and emission spectra. The absorbance and emission spectra are shown in Figure 2b. The black curve displays the UV-vis absorption spectrum of HEFBNPs, indicating that the HEFBNPs were absorbed significantly in the UV region, with maximum absorption at 248 nm. As shown by the red curve, displaying the emission spectrum of HEFBNPs at an excitation wavelength of 248 nm, the HEFBNPs exhibited intense reddish-orange fluorescence with a maximum wavelength of 720 nm. 

HEFBNPs have their own particular fluorescence properties, and the fluorescence intensity of HEFBNPs changes in the presence or absence of H_2_O_2_ (Figure 2c). From a fluorescence perspective, the HEBNPs exhibited no fluorescence (Figure 2c(i)), but the HEFBNPs exhibited strong fluorescence (Figure 2c(ii)), Where the HEBNPs are horseradish peroxidase-encapsulated bio-nanoparticles without gold nanoclusters. This difference was compared with the emission spectra of the HEBNPs and HEFBNPs at an excitation wavelength of 284 nm. To investigate the quenching effect of HEFBNPs in the presence of H_2_O_2_, HEFBNPs were mixed with 100 mM H_2_O_2_ (final concentration = 50 mM). The result showed that the fluorescence intensity of HEFBNPs containing 100 mM of H_2_O_2_ was significantly reduced and visual detection was also possible in Figure 2c(iii).

To demonstrate the outstanding optical properties of HEFBNPs, the fluorescence quenching effect of HEFBNPs was compared with that of BSA-AuNCs, HRP-AuNCs, and a mixture of BSA-AuNCs and HRP-AuNCs in the presence of 100 μM H_2_O_2_ (final concentration = 50 μM). To compare the quenching effect of HEFBNPs and protein-AuNCs in the presence of H_2_O_2_, the normalized fluorescence intensity (F/F_0_) was investigated. The F_0_ is the fluorescence intensity without H_2_O_2_ and the F is as the fluorescence intensity in the presence of H_2_O_2_. As shown in Figure 2d, the HEFBNPs were significantly quenched, whereas BSA-AuNCs, HRP-AuNCs, and a mixture of BSA-AuNCs and HRP-AuNCs exhibited a negligibly decreased fluorescence. The results showed that the fluorescence-quenching effect of the HEFBNPs, caused by a continuous two-step quenching mechanism, was considerable. The fluorescence quenching mechanism can be explained by Equations (1) and (2).

The fluorescence quenching mechanism of HEFBNPs in the presence of H_2_O_2_ is given as follows:

HRP-AuNC (Au^0^/Au^+^) + H_2_O_2_ → HRP-AuNC (Au) (fluorescence quenching) + 2^•^OH, (1)

[2RS-Au → RS-SR + 2Au] in HRP-AuNCs (AuNC agglomeration and deterioration of the AuNC structure)

AuNC (Au^0^/Au^+^) in BSA and HRP + 2^•^OH → AuNC (Au^+^) (fluorescence quenching) + 1/2O_2_ + H_2_O (2)

The surfaces of the HRP-AuNCs and BSA-AuNCs coexisted with Au^0^ and Au^+^. Fluorescence quenching was generated by the oxidation of Au^0^ to Au^+^ and the deformation of the RS-Au (where R is typically a side chain of an amino acid) bonding in HRP-AuNCs [16,17]. In the presence of H_2_O_2_, the Au-S bonding between HRP residues and gold atoms in HRP-AuNCs could replace the redox reaction that formed the RS-SR and Au agglomeration [15]. As a result, in Equation (1), the fluorescence of AuNCs was quenched in HRP-AuNCs and the hydroxyl radical (^•^OH) was made. HRP, which was used for the stabilization of HRP-AuNCs, maintained its intrinsic catalytic activity, which could yield the hydroxyl radical (^•^OH) by reacting with H_2_O_2_ [30]. The protein-AuNCs can also produce ^•^OH by reacting with H_2_O_2_ [31,32]. However, the catalytic efficiency of the protein-AuNCs was very low compared with that of HRP (Figure S3). ^•^OH had a stronger oxidative power than H_2_O_2_; hence, ^•^OH catalyzed the fluorescence quenching. Considering that the ^•^OH was generated by Equation (1), the Au^0^ in the BSA-AuNCs and HRP-AuNCs rapidly oxidized to Au^+^, with 2^•^OH reduced to O_2_ and H_2_O_2_, as shown in Equation (2). In addition, because ^•^OH could reach adjacent AuNCs quickly due to the proximity of enzyme-HRP AuNCs and AuNCs, the overall reaction event was improved, and the loss of ^•^OH in the solution was reduced. Therefore, Equation (2) progressed more efficiently in HEFBNPs than when the BSA-AuNC and HRP-AuNC mixture was dispersed in the solution. Consequently, the fluorescence of HEFBNPs was strongly quenched by the continuous two-step quenching effect and an effective reaction event compared to the previously reported method (Table 1).

### 3.3. Detection of H_2_O_2_ Based on Fluorescence Quenching of HEFBNPs

Before testing the fluorescence quenching event of HEFBNPs according to different concentrations of H_2_O_2_, the fluorescence quenching effect on temperature and pH was investigated. The temperature increased from 5 °C at 10 °C intervals. The normalized signal F/F_0_ tended to decrease slightly as the temperature increased, but the change was negligible from 5 °C to 25 °C (Figure S4). The pH increased from 2 to 12 at intervals of pH 1. Fluorescence quenching of HEFBNPs occurred under strong acid and strong base conditions. The normalized signal F/F_0_ was almost constant between pH 4 and pH 8 (Figure S5). Considering that river or tap water is in the range of pH 5 to 8 and below 25 °C, it could be confirmed from these results that HEFBNPs can be stably used in a real environment. 

**Figure 3:**
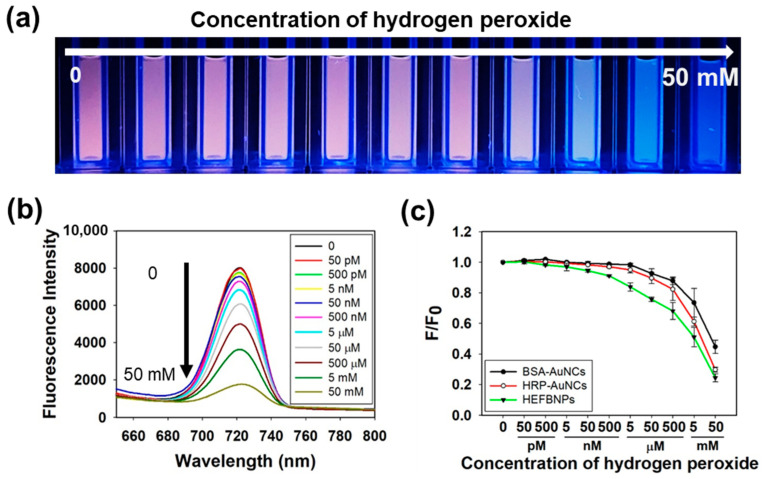
Fluorescence variation according to the various concentration of H_2_O_2_: (**a**) fluorescence image of HEFBNPs under the UV light (H_2_O_2_ concentration: 0, 50 nM, 500 nM, 5 μM, 50 μM, 500 μM, 5 mM, 50 mM), (**b**) fluorescence emission spectra of HEFBNPs, and (**c**) comparison of the fluorescence intensity of BSA-AuNCs, HRP-AuNCs, and HEFBNPs.

The sensitivity of HEFBNPs was tested by investigating the fluorescence variation of HEFBNPs according to different concentrations of H_2_O_2_. The fluorescence intensity was obtained by mixing the HEFBNPs solution with various concentrations of H_2_O_2_ solution (0.0001, 0.001, 0.01, 0.1, 1, 10, 100, 1000, 10,000, and 100,000 μM) in a ratio of 1:1. As a result, the final concentrations of measured H_2_O_2_ were 0.00005, 0.0005, 0.005, 0.05, 0.5, 5, 50, 500, 5000, and 50,000 μM. As shown in Figure 3a, the fluorescence of the HEFBNPs gradually turned off as the concentration of H_2_O_2_ increased under UV light. Therefore, the change in fluorescence could be visually detected up to 50 μM with the naked eye. To detect H_2_O_2_ more accurately and quantitatively, fluorescence spectra were recorded, and the response of HEFBNPs to various concentrations of H_2_O_2_ was represented (Figure 3b). The results showed that the fluorescence intensity of the HEFBNPs decreased as the concentration of H_2_O_2_ increased from 50 pM to 50 mM under an excitation wavelength of 248 nm. A HEFBNP-based sensing system could measure very low concentrations of H_2_O_2_ up to 0.5 nM. Further, the fluorescence quenching effect of HEFBNPs was compared with those of BSA-AuNCs and HRP-AuNCs to demonstrate HEFBNPs’ sensing performance of HEFBNPs. The fluorescence intensity of BSA-AuNCs was recorded at an emission wavelength of 635 nm under an excitation wavelength of 290 nm. That of HRP-AuNCs was recorded at an emission wavelength of 750 nm under an excitation wavelength of 260 nm. As shown in Figure 3c, the fluorescence quenching effects of HEFBNPs and protein-AuNCs were compared using the normalized fluorescence intensity (F/F_0_). The results showed that the fluorescence intensity of the BSA-AuNCs and HRP-AuNCs strongly decreased from 50 μM to 50 mM. However, the fluorescence of HEFBNPs drastically decreased starting from very low concentration ranges from 50 nM to 50 mM, and showed significantly decreased fluorescence compared to BSA-AuNCs and HRP-AuNCs at the same concentration of H_2_O_2_. In addition, the detection limits of BSA-AuNCs and HRP-AuNCs were approximately 5 μM and 50 nM, respectively, whereas that of HEFBNPs was 0.5 nM. Therefore, HEFBNPs showed a high sensitivity for the detection of H_2_O_2_.

**Figure 4:**
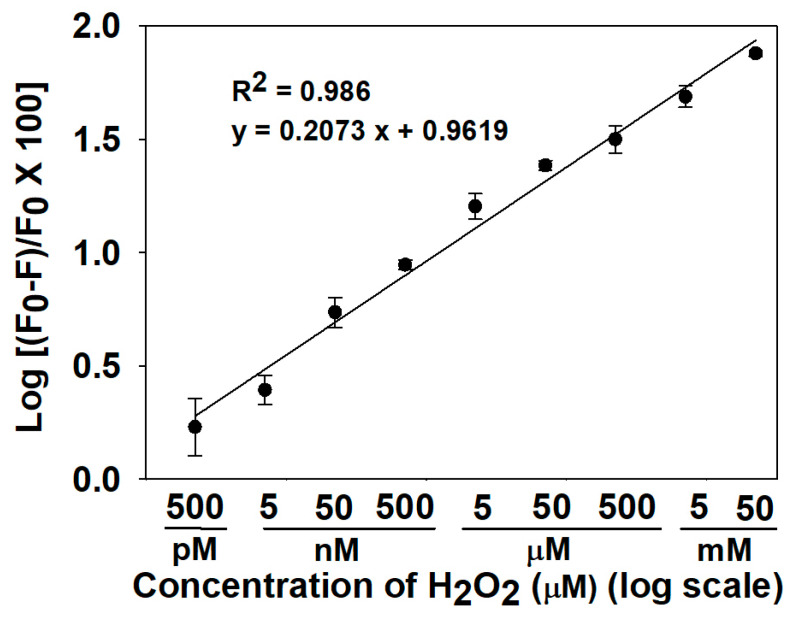
Linear relation between the logarithmic value of fluorescence quenching ratio and the value of H_2_O_2_ concentration from 500 pM to 50 mM.

The calibration curve based on the fluorescence quenching effect of HEFBNPs according to different concentrations of H_2_O_2_ is shown in Figure 4. We plotted the calibration curve of the value of (F_0_ − F)/F_0_ × 100 (%) versus the concentration of H_2_O_2_ (both values are log scale). The fluorescence quenching was linearly related to H_2_O_2_ with high linearity (R^2^ = 0.986) in wide range of concentrations of H_2_O_2_ from 500 pM to 50 mM. From this result, it could be claimed that our HEFBNP-based sensing system has a good quantitative relationship to determine the accurate concentration of H_2_O_2_. 

### 3.4. Selectivity of HEFBNPs-Based Fluorescence Sensor for H_2_O_2_

**Figure 5:**
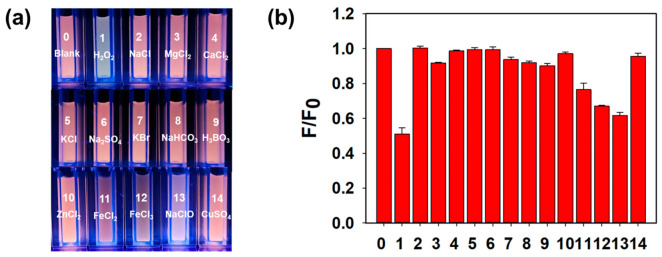
Selectivity test of HEFBNPs for H_2_O_2_ compared to nontarget materials: (**a**) image of HEFBNPs under the UV light after addition of different materials and (**b**) normalized fluorescence intensity ratio (F/F_0_) after adding different materials (0: Blank, 1: H_2_O_2_, 2: NaCl, 3: MgCl_2_, 4: CaCl_2_, 5: KCl, 6: Na_2_SO_4_, 7: KBr, 8: NaHCO_3_, 9: H_3_BO_3,_ 10: ZnCl_2_, 11: FeCl_2_, 12: FeCl_3_, 13: NaClO, and 14: CuSO_4_).

HEFBNPs selectively detected H_2_O_2_ because they contained the enzyme HRP that could specifically react with H_2_O_2_. To verify the specificity of HEFBNPs for H_2_O_2_, other components were employed as non-target materials such as metal-salt and oxidizing substances including NaCl, MgCl_2_, CaCl_2_, KCl, Na_2_SO_4_, KBr, NaHCO_3_, HBO_3_, ZnCl_2_, FeCl_2_, FeCl_3_, and NaClO (Figure 5a,b). The different components of solution and H_2_O_2_ were separately diluted in DW to a concentration of 10 mM. Each solution was added to HEFBNPs at a ratio of 1:1 (final analyte concentration = 5 mM). Photographs of fluorescence quenching after adding non-target materials and H_2_O_2_ are shown in Figure 5a. The fluorescence of the HEFBNP solution was quenched by H_2_O_2_ and the color change could be detected with the naked eye. The fluorescence color of the HEFBNP solution by the non-target materials remained unchanged, but the oxidizing substances such as FeCl_2_, FeCl_3_, and NaClO changed the fluorescence. For a more accurate evaluation, F/F_0_ was recorded for the HEFBNP solution containing H_2_O_2_ or each nontarget material (Figure 5b). As a result, H_2_O_2_ significantly decreased the fluorescence intensity of HEFBNPs, whereas there was no obvious change when mixed with NaCl, MgCl_2_, CaCl_2_, KCl, Na_2_SO_4_, KBr, NaHCO_3_, HBO_3_, and ZnCl_2_. However, when mixed with an oxidizing substance such as Fe^2+^ or Fe^3+^, the HEFBNPs’ fluorescence intensity was decreased. This phenomenon comes from the quenching of AuNCs regardless of the ligand. Many studies showed that Fe^2+^ and Fe^3+^ could suppress the fluorescence of BSA-AuNCs. Fe^3+^ was reported to be able to aggregate AuNCs, which is probably due to the interaction between Fe^3+^ and the residual carboxylate group of ligands [38,39]. The aggregated AuNCs do not show optical features of nanoclusters that show fluorescence. Since Fe^2+^ can be oxidized to Fe^3+^, the fluorescence of AuNCs can be quenched by Fe^2+^, but its affect is not critical compared to Fe^3+^ [40]. As shown in Figure 5b, Fe^3+^ and Fe^2+^ decreased fluorescence intensity and the fluorescence quenching effect of Fe^2+^ was not higher than that of Fe^3+^. ClO^-^ is one of the types of ROS that can decrease the fluorescence intensity of AuNCs [41]. ClO^-^ can quench the fluorescence of AuNCs by acting on HEFBNPs in the same way as ^•^OH. However, the degree of the decrease in fluorescence intensity by ClO^-^ was less affected than that of H_2_O_2_. This is because H_2_O_2_ can cause additional fluorescence quenching by the reaction of HRP and AuNCs, but ClO^-^ acts only on AuNCs, causing quenching. Despite the high concentration of nontarget materials, the materials did not affect the detection results as much as H_2_O_2_. Therefore, it could be confirmed that the detection system for H_2_O_2_ showed good selectivity.

To investigate the performance of HEFBNPs in a real sample, we analyzed a river water sample from ‘Han River’ (Figure S6). The calculated accuracy, recovery, and precision with three concentrations of H_2_O_2_ in river water were 75.1%, 124.9%, and 9.44%, respectively. When comparing the analysis value of quenching ratio = (F_0_ − F)/F_0_ of river water with the value of the calibration curve (concentration of H_2_O_2_ fixed), it could be confirmed that the analysis data using river water were also suitable for our detection system.

### 3.5. Glass-Based Microfluidic Device for Detection of H_2_O_2_

**Figure 6:**
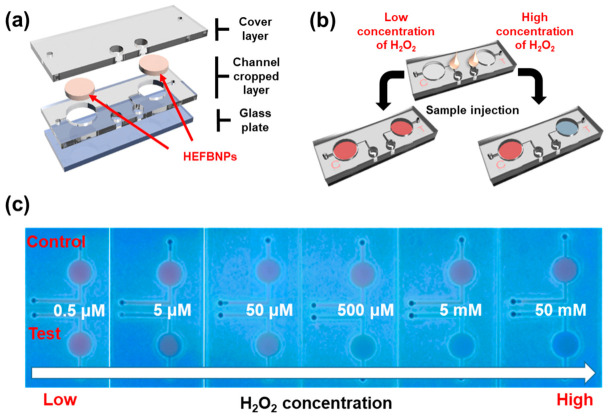
Schematic representation of (**a**) structure of the glass-based microfluidic device and (**b**) H_2_O_2_ detection using a glass-based microfluidic device. (**c**) Image of the fluorescence change of HEFBNPs in a glass-based microfluidic device after adding different concentrations of H_2_O_2_ under UV light.

To simplify the use of HEFBNPs, we fabricated a glass-based microfluidic device to measure H_2_O_2_. This device efficiently detects H_2_O_2_ because the reaction time of HEFBNPs and H_2_O_2_ is as short as 10 min, and the fluorescence change of HEFBNPs can be seen directly on-site using a portable UV illuminator. The device was made by stacking layers of a glass plate, channel-cropped acrylic plate, and cover acrylic plate (Figure 6a). 

The device has two microfluidic channels: control and test channels. HEFBNPs were immobilized on a glass surface at the signal pot in each channel. After DW and different concentrations of H_2_O_2_ were added to the control and test lines, respectively, the device was placed under UV light to investigate the fluorescence of HEFBNPs (Figure 6b). At the control line signal pot, no quenching effect occurred. The original fluorescence intensity and color of HEFBNPs were observed. Contrarily, fluorescence quenching occurred at the signal pot in the test line according to the concentration of H_2_O_2_, as previously shown in this paper. By comparing the fluorescence intensity or color of the control and test signal pots, the concentration of H_2_O_2_ in the substrate was analyzed. As shown in Figure 6c, the fluorescence color change according to the concentration of H_2_O_2_ could be detected by the naked eye. Overall, these simple detection methods and lightweight devices are expected to be suitable for the point-of-care detection of H_2_O_2_.

## 4. Conclusions

In this study, we developed HEFBNPs with fluorescence properties for the sensitive detection of H_2_O_2_ based on the fluorescence quenching of HEFBNPs. Compared with BSA-AuNCs and HRP-AuNCs, HEFBNPs could detect H_2_O_2_ more sensitively because of their continuous two-step fluorescence quenching effect and improved reaction event. As a result, HEFBNPs could detect a wide concentration range of H_2_O_2_ up to a low concentration (0.5 nM) with good linearity and selectivity. For the easy use of HEFBNPs, we fabricated a glass-based microfluidic device and measured the concentration of H_2_O_2_ by the naked eye using the fluorescence changes of HEFBNPs. Owing to the simple detection method and portability of the device, HEFBNPs are suitable for use in POCTs. Moreover, the proposed sensing system can be used to detect other substances, such as glucose, lactate, and uric acid, using enzymes that can generate H_2_O_2_. In conclusion, we expect that our proposed HEFBNP-based fluorescence sensing system is expected to be an easy and highly sensitive on-site detection tool in chemistry, biology, clinics, and industry fields.

## Figures and Tables

**Table 1:** Comparison with previously reported fluorescence detection methods of H_2_O_2_.

Fluorescence Probe	Analytical Range (μM)	Detection Limit (μM)	Reference
BSA-CuNCs	0–100	0.082	[33]
GOx-AuNCs	0.5–10	0.23	[34]
PEI-AgNCs	0.5–10	0.1	[35]
BSA-AuNCs	1–50,000	0.7	[14]
HRP-AuNCs	0.1–100	0.03	[15]
CdSe@ZnS/AgNCs	0–100	0.3	[36]
Si-O QDs/AgNCs	8–60,000	6.5	[37]
HEFBNPs	0.0005–50,000	0.0005	Present work

## Data Availability

Not applicable.

## Supplementary Materials

The following supporting information can be downloaded at: https://www.mdpi.com/article/10.3390/bios13020289/s1. Figure S1. Synthesis procedure of HEFBNPs by desolvation and cross-linking methods; Figure S2. Encapsulated HRP-AuNC within HEFBNP via crosslinking process with HRP-AuNC and BSA-AuNC mixture; Figure S3. Comparison enzymatic reaction of materials in HEFBNPs (n = 3); Figure S4. Influence of temperature on the detection of H_2_O_2_ using HEFBNPs (n = 3); Figure S5. Influence of pH on the detection of H_2_O_2_ using HEFBNPs (n = 3); Figure S6. Comparison of results from HEFBNPs analysis using real river water with calibration curve that conducted by experimental condition (n = 4). When the real sample analyzed, the condition as follows; 20 °C, pH 7, F_0_ = Fluorescence intensity when HEFBNPs mixed with blank river water, F = Fluorescence intensity when HEFBNPs mixed with river water containing known concentration of H_2_O_2_. River water samples containing known concentrations of H_2_O_2_ were artificially made with final concentration of H_2_O_2_ = 50, 100, 200 μM using blank river water. The fluorescence analyzing method of F and F_0_ were same as manuscript.

## References

[1] Chen H.Y., Wu L., Wan Y.Q., Huang L.L., Li N.X., Chen J.Y. (2019). One-step rapid synthesis of fluorescent silicon nanodots for a hydrogen peroxide-related sensitive and versatile assay based on the inner filter effect. Analysts.

[2] Ye S., Hananya N., Green O., Chen H.S., Zhao A.Q., Shen J.G. (2020). A Highly Selective and Sensitive Chemiluminescent Probe for Real-Time Monitoring of Hydrogen Peroxide in Cells and Animals. Angew. Chem. Int. Ed..

[3] Liu J.Y., Qin Y.N., Li D., Wang T.S., Liu Y.Q., Wang J. (2013). Highly sensitive and selective detection of cancer cell with a label-free electrochemical cytosensor. Biosens. Bioelectron..

[4] Giorgio M., Trinei M., Migliaccio E., Pelicci P.G. (2007). Hydrogen peroxide: A metabolic by-product or a common mediator of aging signals?. Nat. Rev. Mol. Cell Biol..

[5] Hopwood M.J., Rapp I., Schlosser C., Achterberg E.P. (2017). Hydrogen peroxide in deep waters from the Mediterranean Sea, South Atlantic and South Pacific Oceans. Sci. Rep..

[6] Ning K.K., Xiang G.Q., Wang C.C., Huang F.H., Liu J.Z., Zhang L.L. (2020). ‘Turn-on’ fluorescence sensing of hydrogen peroxide in marine food samples using a carbon dots-MnO_2_ probe. Luminescence.

[7] Arulraj A.D., Devasenathipathy R., Chen S.-M., Vasantha V.S., Wang S.-F. (2015). Highly selective and sensitive fluorescent chemosensor for femtomolar detection of silver ion in aqueous medium. Sens. Bio-Sens. Res..

[8] Li H.L., Zhu W.L., Wan A.J., Liu L.B. (2017). The mechanism and application of the protein-stabilized gold nanocluster sensing system. Analyst.

[9] Cui H., Shao Z.S., Song Z., Wang Y.B., Wang H.S. (2020). Development of gold nanoclusters: From preparation to applications in the field of biomedicine. J. Mater. Chem. C.

[10] Goswami N., Zheng K.Y., Xie J.P. (2014). Bio-NCs-the marriage of ultrasmall metal nanoclusters with biomolecules. Nanoscale.

[11] Govindaraju S., Ankireddy S.R., Viswanath B., Kim J., Yun K. (2017). Fluorescent Gold Nanoclusters for Selective Detection of Dopamine in Cerebrospinal fluid. Sci. Rep..

[12] Li C.G., Chen H., Chen B., Zhao G.H. (2018). Highly fluorescent gold nanoclusters stabilized by food proteins: From preparation to application in detection of food contaminants and bioactive nutrients. Crit. Rev. Food Sci. Nutr..

[13] Deng H.H., Wu G.W., He D., Peng H.P., Liu A.L., Xia X.H. (2015). Fenton reaction-mediated fluorescence quenching of N-acetyl-L-cysteine-protected gold nanoclusters: Analytical applications of hydrogen peroxide, glucose, and catalase detection. Analysts.

[14] Jain V., Bhagat S., Singh S. (2021). Bovine serum albumin decorated gold nanoclusters: A fluorescence-based nanoprobe for detection of intracellular hydrogen peroxide. Sens. Actuators B Chem..

[15] Wen F., Dong Y.H., Feng L., Wang S., Zhang S.C., Zhang X.R. (2011). Horseradish Peroxidase Functionalized Fluorescent Gold Nanoclusters for Hydrogen Peroxide Sensing. Anal. Chem..

[16] Chen Y.Y., Zhong Q.M., Wang Y.L., Yuan C.L., Qin X., Xu Y.J. (2019). Colorimetric detection of hydrogen peroxide and glucose by exploiting the peroxidase-like activity of papain. RSC Adv..

[17] Shen R., Liu P.P., Zhang Y.Q., Yu Z., Chen X.Y., Zhou L. (2018). Sensitive Detection of Single-Cell Secreted H_2_O_2_ by Integrating a. Microfluidic Droplet Sensor and Au Nanoclusters. Anal. Chem..

[18] Yang D.Q., Luo M.C., Di J.W., Tu Y.F., Yan J.L. (2018). Gold nanocluster-based ratiometric fluorescent probes for hydrogen peroxide and enzymatic sensing of uric acid. Microchim. Acta..

[19] Mi W.Y., Tang S., Jin Y., Shao N. (2021). Au/Ag Bimetallic Nanoclusters Stabilized by Glutathione and Lysozyme for Ratiometric Sensing of H_2_O_2_ and Hydroxyl Radicals. ACS Appl. Nano Mater..

[20] Nguyen L.T., Yang K.L. (2017). Combined cross-linked enzyme aggregates of horseradish peroxidase and glucose oxidase for catalyzing cascade chemical reactions. Enzym. Microb. Technol..

[21] Xie J.P., Zheng Y.G., Ying J.Y. (2009). Protein-Directed Synthesis of Highly Fluorescent Gold Nanoclusters. J. Am. Chem. Soc..

[22] Yan L., Cai Y.Q., Zheng B.Z., Yuan H.Y., Guo Y., Xiao D. (2012). Microwave-assisted synthesis of BSA-stabilized and HSA-protected gold nanoclusters with red emission. J. Mater. Chem..

[23] Choi J.H., Lim Y.T., Oh B.K. (2014). Development of Colorimetric Enzyme-Ball for Signal Amplification of Enzyme-Linked Immunosorbent Assay. Sci. Adv. Mater..

[24] Hong S., Choi D.W., Kim H.N., Park C.G., Lee W., Park H.H. (2020). Protein-Based Nanoparticles as Drug Delivery Systems. Pharmaceutics.

[25] Elzoghby A.O., Samy W.M., Elgindy N.A. (2012). Albumin-based nanoparticles as potential controlled release drug delivery systems. J. Control. Release.

[26] Li F.Q., Su H., Wang J., Liu J.Y., Zhu Q.G., Fei Y.B., Pan Y.H., Hu J.H. (2008). Preparation and characterization of sodium ferulate entrapped bovine serum albumin nanoparticles for liver targeting. Int. J. Pharm..

[27] Migneault I., Dartiguenave C., Bertrand M.J., Waldron K.C. (2004). Glutaraldehyde: Behavior in aqueous solution, reaction with proteins, and application to enzyme crosslinking. Biotechniques.

[28] Yu Z., Yu M., Zhang Z., Hong G., Xiong Q. (2014). Bovine serum albumin nanoparticles as controlled release carrier for local drug delivery to the inner ear. Nanoscale Res. Lett..

[29] Shin J.H., Lee M.J., Choi J.H., Song J.A., Kim T.H., Oh B.K. (2020). Electrochemical H_2_O_2_ biosensor based on horseradish peroxidase encapsulated protein nanoparticles with reduced graphene oxide-modified gold electrode. Nano Converg..

[30] Huang X., Groves J.T. (2018). Oxygen Activation and Radical Transformations in Heme Proteins and Metalloporphyrins. Chem. Rev..

[31] Chen T., Hu Y., Cen Y., Chu X., Lu Y. (2013). A Dual-Emission Fluorescent Nanocomplex of Gold-Cluster-Decorated Silica Particles for Live Cell Imaging of Highly Reactive Oxygen Species. J. Am. Chem. Soc..

[32] Yang S., Jiang Z., Chen Z., Tong L., Lu J., Wang J. (2015). Bovine serum albumin-stabilized gold nanoclusters as a fluorescent probe for determination of ferrous ion in cerebrospinal fluids via the Fenton reaction. Microchim. Acta..

[33] Wang C.J., Yang M., Mi G.H., Zhang B., Dou X.H., Liu E.Z., Hu X.Y., Xue W.M., Fan J. (2021). Dual-emission fluorescence sensor based on biocompatible bovine serum albumin stabilized copper nanoclusters for ratio and visualization detection of hydrogen peroxide. Dye. Pigm..

[34] Cui W.W., Qin H.Y., Zhou Y., Du J.X. (2017). Determination of the activity of hydrogen peroxide scavenging by using blue-emitting glucose oxidase-stabilized gold nanoclusters as fluorescent nanoprobes and a Fenton reaction that induces fluorescence quenching. Microchim. Acta..

[35] Wen T., Qu F., Li N.B., Luo H.Q. (2012). Polyethyleneimine-capped silver nanoclusters as a fluorescence probe for sensitive detection of hydrogen peroxide and glucose. Anal. Chim. Acta..

[36] Zhou Z.Q., Yang L.Y., Huang L., Liao Y.P., Liu Y., Xiao Q. (2020). A novel fluorescent probe for H_2_O_2_ detection based on CdSe@ZnS quantum dots/Ag nanocluster hybrid. Anal. Chim. Acta..

[37] Dong R.Y., Yao Y.Y., Li D.N., Zhang H.R., Li W., Molokee M. (2020). Ratio fluorescent hybrid probe for visualized fluorescence detection of H_2_O_2_ in vitro and in vivo. Sens. Actuators B Chem..

[38] Deng H.H., Huang K.Y., Zhang M.J., Zou Z.Y., Xu Y.Y., Peng H.P., Chen W., Hong G.L. (2020). Sensitive and selective nitrite assay based on fluorescent gold nanoclusters and Fe^2+^/Fe^3+^ redox reaction. Food Chem..

[39] Huang H., Li H., Feng J.J., Wang A.J. (2016). One-step green synthesis of fluorescent bimetallic Au/Ag nanoclusters for temperature sensing and in vitro detection of Fe^3+^. Sens. Actuators B Chem..

[40] Sebastian A., Aarya, Sarangi B.R., Sen Mojumdar S. (2023). Lysozyme protected copper nano-cluster: A photo-switch for the selective sensing of Fe^2+^. J. Photochem. Photobiol. A.

[41] Quan Z.Y., Xue F., Li H.Y., Chen Z.P., Wang L., Zhu H.X., Pang C.L., He H. (2022). A bioinspired ratiometric fluorescence probe based on cellulose nanocrystal-stabilized gold nanoclusters for live-cell and zebrafish imaging of highly reactive oxygen species. Chem. Eng. J..

