# Supplementary material for: Horseradish Peroxidase-Encapsulated Fluorescent Bio-Nanoparticle for Ultra-Sensitive and Easy Detection of Hydrogen Peroxide
Source: Biosensors (Basel). 2023 Feb 17;13(2):289. doi: 10.3390/bios13020289 (PMC9953809; doi:10.3390/bios13020289)
Supplement: Supplementary file 1 [file biosensors-13-00289-s001.zip › biosensors-2159584-supplementary.pdf]

biosensors

Supporting Information

Horseradish Peroxidase-Encapsulated Fluorescent Bio-Nanoparticle for Ultra-Sensitive and Easy Detection of Hydrogen Peroxide

Myeong-Jun Lee[1,†], Ji-Ae Song[1,†], Jin-Ha Choi[2], Jeong-Hyeop Shin[1], Ji-Woon Myeong[1], Ki-Ppeum Lee[1], Taehwan Kim[1], Ki-Eob Park[3] and Byung-Keun Oh[1,*]

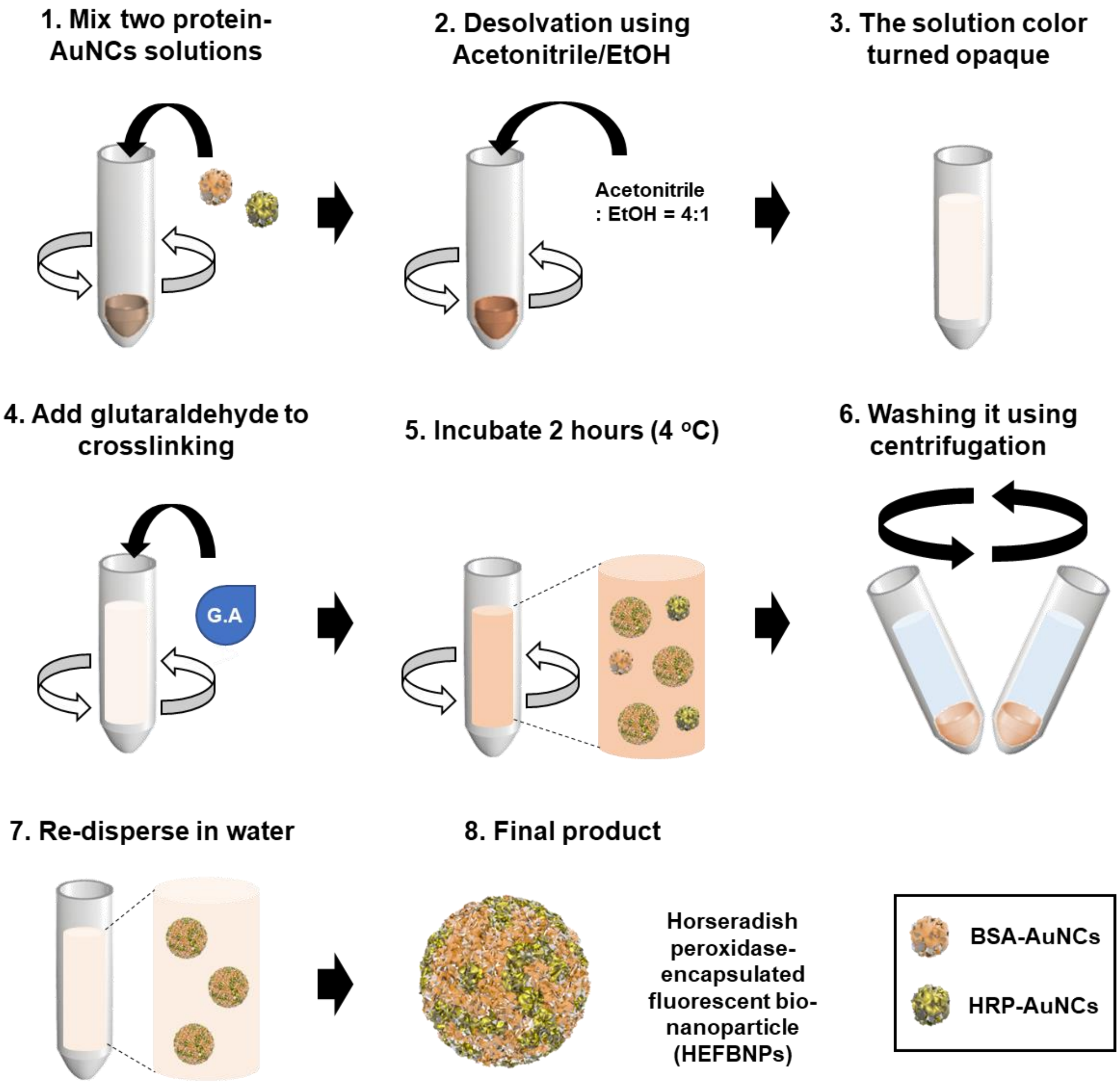

Figure S1. Synthesis procedure of HEFBNPs by desolvation and cross-linking methods.

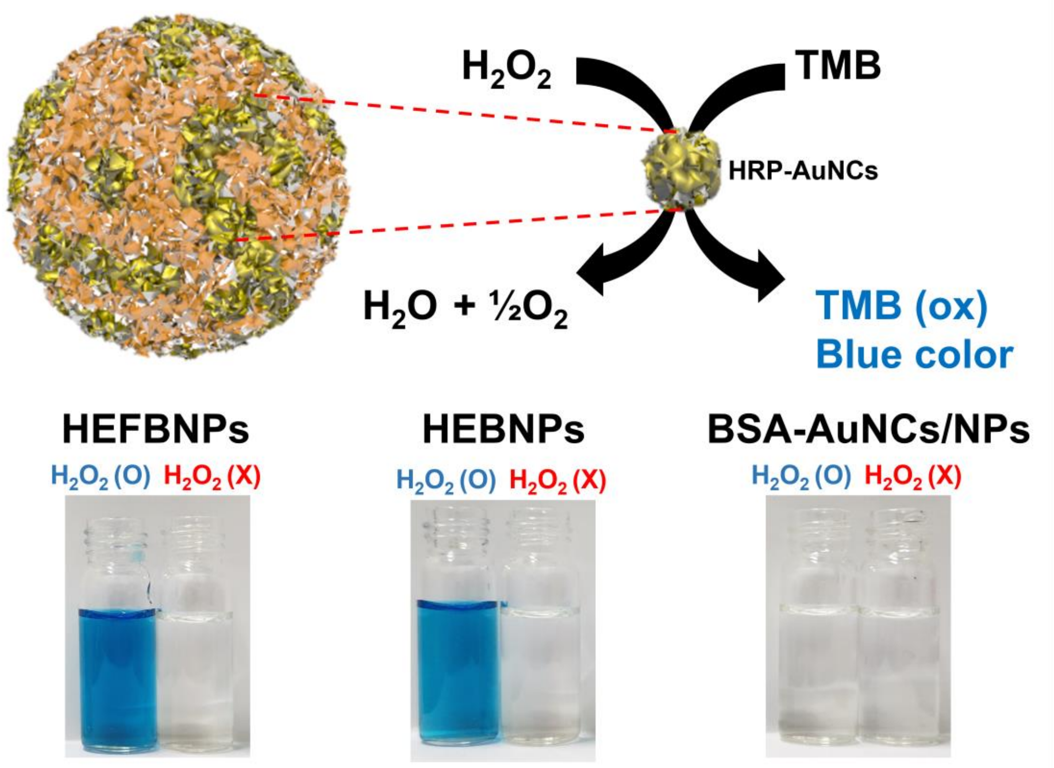

Figure S2. Encapsulated HRP-AuNC within HEFBNP via crosslinking process with HRP-AuNC and BSA-AuNC mixture.

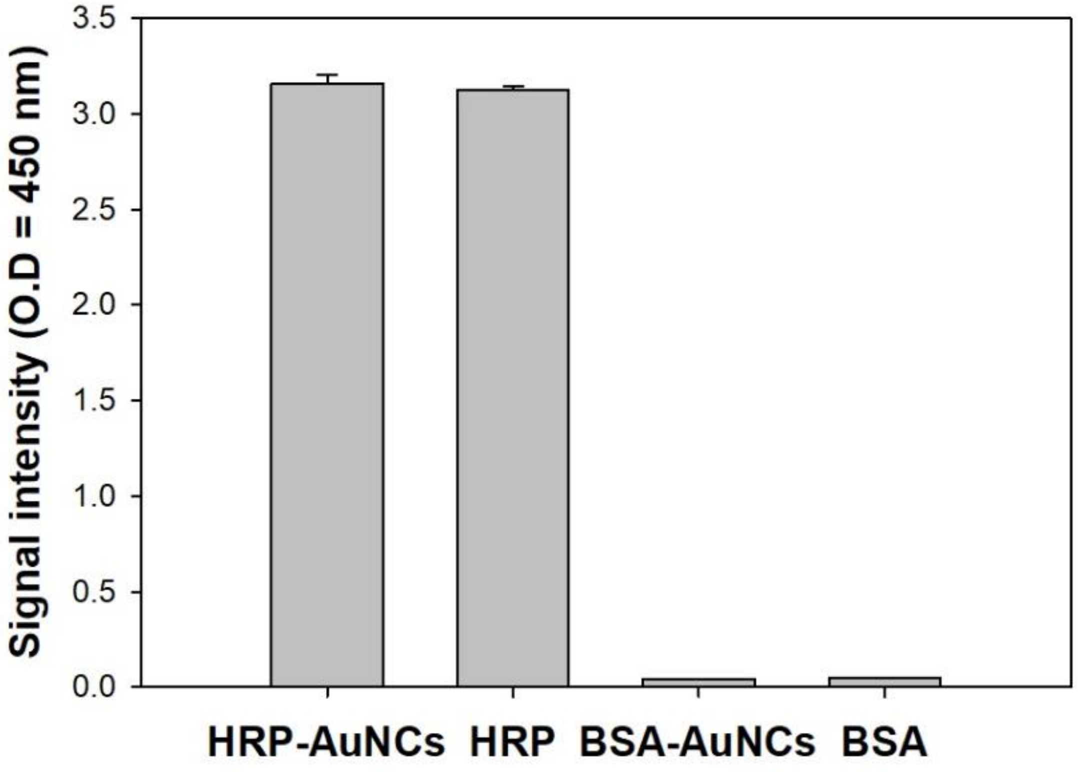

Figure S3. Comparison enzymatic reaction of materials in HEFBNPs (n=3).

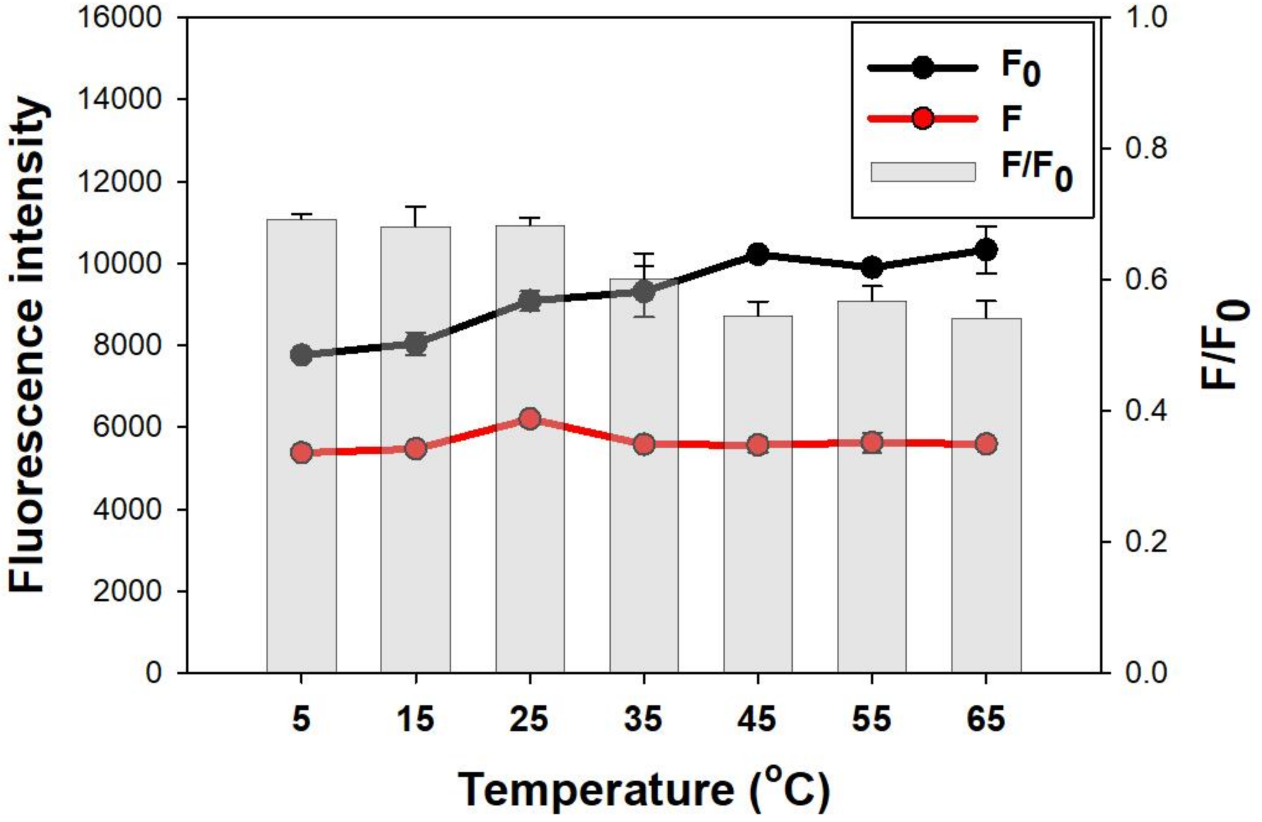

Figure S4. Influence of temperature on the detection of H_2O_2 using HEFBNPs (n=3).

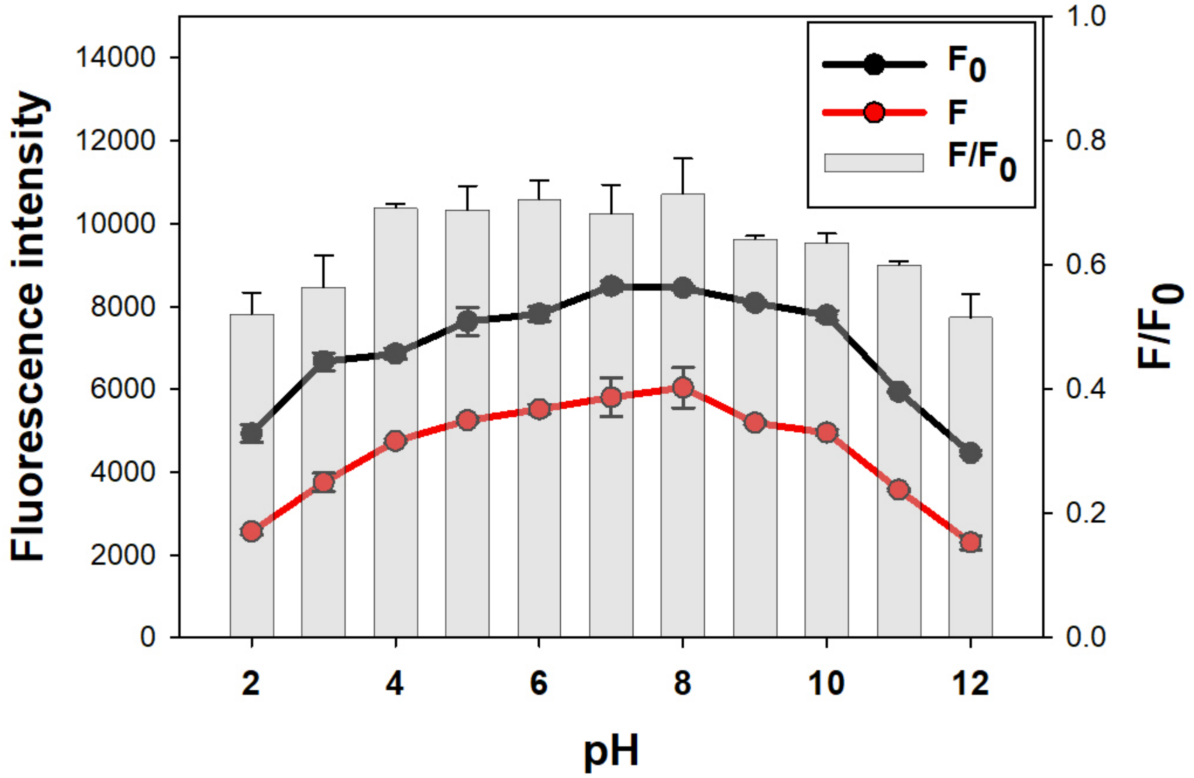

Figure S5. Influence of pH on the detection of H_2O_2 using HEFBNPs (n=3).

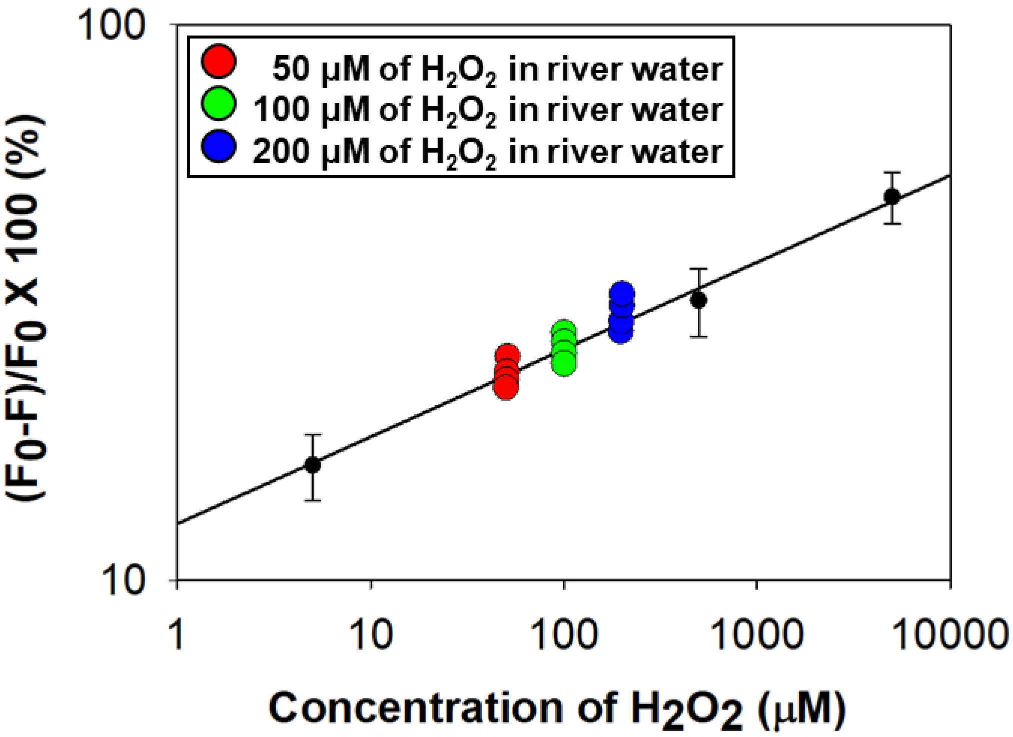

Figure S6. Comparison of results from HEFBNPs analysis using real river water with calibration curve that conducted by experimental condition (n=4). When the real sample analyzed, the condition as follows; 20 °C, pH 7, F_0 = Fluorescence intensity when HEFBNPs mixed with blank river water, F = Fluorescence intensity when HEFBNPs mixed with river water containing known concentration of H_2O_2. River water samples containing known concentrations of H_2O_2 were artificially made with final concentration of H_2O_2 = 50, 100, 200 μM using blank river water. The fluorescence analyzing method of F and F_0 were same as manuscript.
